# Outcome of keratolimbal allograft transplantation with deep anterior lamellar keratoplasty for bilateral limbal stem cell deficiency

**DOI:** 10.3389/fmed.2022.986194

**Published:** 2022-11-15

**Authors:** Zongyuan Li, Kunkun Yang, Yannan Zhou, Tengyun Wu, Hongtao Zhang, Qinghua Yang, Qun Wang, Yifei Huang, Liqiang Wang

**Affiliations:** ^1^Medical School of Chinese PLA, Beijing, China; ^2^Department of Ophthalmology, The First Medical Center, Chinese PLA General Hospital, Beijing, China; ^3^Senior Department of Ophthalmology, The Third Medical Center, Chinese PLA General Hospital, Beijing, China

**Keywords:** keratolimbal allograft, limbal stem cell deficiency, limbus, cornea, deep anterior lamellar keratoplasty, survival analysis, visual acuity

## Abstract

**Objectives:**

To evaluate and compare the outcome of keratolimbal allograft (KLAL) transplantation with or without deep anterior lamellar keratoplasty (DALK) for bilateral severe limbal stem cell deficiency (LSCD).

**Methods:**

This retrospective review included 49 eyes of 46 patients who underwent KLAL transplantation at the Department of Ophthalmology of Chinese PLA general hospital, 2009–2020, for bilateral severe LSCD were examined for corneal clarity and corneal scarring to determine whether to combine DALK with KLAL transplantation. Preoperative information, surgical decision tree, surgical procedures, and postoperative data were collected for each eye.

**Results:**

All patients had preoperative severe or total LSCD. Twenty-four eyes underwent KLAL transplantation only, 25 KLAL transplantation plus DALK. The mean follow-up was 46.80 ± 31.22 months (18–158 months). Overall KLAL survival (with or without DALK) was 71.43% at the final follow-up (KLAL-only 66.67%, KLAL-DALK 76%). Kaplan–Meier survival analysis showed that the 3-year survival probability of all grafts was 70.53 ± 10.89% (KLAL-only 64.86 ± 10.11%, KLAL-DALK 75.79 ± 8.62%). The proportion of BCVA ≥ 20/200 eyes among all KLAL transplantations increased from 11 eyes (22.45%) preoperatively to 25 eyes (51.02%) after 1 year and 24 eyes (48.98%) at the last follow-up (*P* = 0.01). The proportion of BCVA ≥ 20/200 eyes in the KLAL-DALK group increased significantly (*P* = 0.04), from 16.0% at baseline to 48.0% after 1 year to 44.0% at the last follow-up. Seventeen eyes (34.69%) had postoperative complications.

**Conclusion:**

KLAL-DALK is an effective option to restore a stable ocular surface and visual acuity rapidly in patients with bilateral, late-stage, severe LSCD.

## Introduction

The corneal epithelium is of a non-keratinizing stratified squamous type, and a healthy epithelium is vital to the preservation of corneal transparency. Corneal epithelial stem cells are adult somatic stem cells located in the limbus that replenish the loss of corneal epithelial cells in both health and disease (1). When these limbal stem cells become dysfunctional or deficient, limbal stem cell deficiency (LSCD) develops (2). LSCD is a major cause of corneal scarring and is particularly prevalent after chemical and thermal burns and immune-mediated diseases of the ocular surface. LSCD causes the limbus to lose its barrier function and conjunctival epithelium growth over the corneal surface, eventually leading to conjunctivalization, neovascularization, recurrent or persistent epithelial defects, ocular surface inflammation, and scarring, which lead to decreased vision, pain, and impaired quality of life (3, 4). When total LSCD involves only one eye or bilateral LSCD exists with spared portions of the healthy limbus, successful reconstruction can be achieved by transplanting autologous limbal epithelial stem cells from the contralateral eye or from the healthy limbal tissue (2, 5, 6). However, in bilateral total LSCD, in which the limbus is completely destroyed in both eyes, limbal tissue from a deceased donor, a living relative, or an alternative source of stem cells can be used for allotransplantation (7). The use of alternative sources of ectopic stem cells, such as the oral mucosa, bone-marrow-derived mesenchymal stem cells, or iPS-cells, is under evaluation as an alternative to limbal allografts obtained from cadavers or living relatives lacking reports of long-term stable efficacy (6, 8–10). In the case of bilateral total LSCD or unilateral total LSCD without a healthy contralateral donor eye, the transplantation of allogeneic limbal stem cells is the only alternative for successful corneal surface restoration (2, 5, 6, 11). This can be achieved with KLALs from cadaveric donors (5, 12, 13) or by limbal conjunctival allografts from living related donors (lr-CLALs) (12–14). Success rates seem to be better with KLALs than with lr-CLALs, and the reported long-term success rates in terms of the stabilization of the ocular surface range between 27 and 72% (15–17). A KLAL is a 360-degree lamellar ring graft encompassing a minimal portion of scleral tissue, the entire limbus, the outermost portion of the cornea, and more limbal stem cells that penetrating keratoplasty (PKP) or lamellar keratoplasty (LKP) cannot supply (13). In some cases, KLAL transplantation requires an additional, simultaneous or sequential, PKP to restore corneal transparency when LSCD is accompanied by deep corneal scarring. However, allograft transplantation performed simultaneously with PKP has fared worse, and the survival of these PKP grafts has been low (16, 18). Simultaneous deep anterior lamellar keratoplasty (DALK) combined with KLAL transplantation is another alternative that was first reported by Tsubota et al. (19). They investigated the incidence and prognosis of immunologic rejection of the central graft after surgery but did not mention its long-term outcomes or complications (19). Whether DALK combined with KLAL transplantation is an effective treatment option to restore a stable ocular surface in patients with bilateral severe LSCD in the long term remains unknown. In this study, we evaluated the role of DALK combined with KLAL transplantation in the treatment of severe LSCD and compared the therapeutic outcomes with those of KLAL transplantation only.

## Methods and procedures

This was a retrospective, non-comparative, interventional case series that included 46 eyes of 49 patients who met the inclusion criteria and were included for analysis between January 2009 and October 2020. The last follow-up visits for all patients, for the purpose of data analysis, took place between February and March 2022. The mean follow-up period was 46.80 ± 31.22 months (range 18–158 months).

### Patients

According to local law, no ethical approval was required for this retrospective analysis. Each patient gave written informed consent after being explained the nature, risks, and possible adverse consequences of the procedure. The inclusion criteria for the study were patients with bilateral severe (or total) LSCD, compensable corneal endothelial function, good tear film function and good eyelid closure who underwent KLAL transplantation with or without DALK, had been followed up for at least 12 months postoperatively and received systemic immunosuppression. The stage of LSCD in all patients was defined based on the most recent global consensus (7) (Supplementary material 1). No eyes presented chronic conjunctival inflammation before surgery, which is a poor prognosis.

The primary outcome measure was the success rate of treatment. The treatment was considered successful if all symptoms had disappeared and a transparent, avascular, and stable corneal surface had been restored (20). The treatment was considered partially successful if the stable corneal surface had been restored and most symptoms had disappeared but superficial neovascularization had recurred, even if it was not as extensive as at the time of admission (20). Treatment failure was defined as the presence of symptoms, recurrent epithelial defects, fleshy fibrovascular pannus, or corneal perforation at the last follow-up (20). Secondary outcome measures were best-corrected visual acuity (BCVA) and the incidence of postoperative complications due to surgery or medical therapy. Patient characteristics are presented in Table 1.

**TABLE 1:** Baseline characteristics of patients who underwent KLAL transplantation.

Characteristics	KLAL all eyes	KLAL- only	KLAL-DALK	*P*-value
**Demographics**				
No. patients	47	24	23	
No. eyes	49	24	25	
Mean age at time of surgery (SD)	39.8 ± 12.8	40.6 ± 14.0	38.1 ± 11.2	
No. Male (%)	41 (87.2)	22 (91.7)	21 (84.0)	*P* = 0.67
No. Female (%)	6 (12.8)	2 (8.3)	4 (16.0)	
**Postoperative follow-up duration (mos)**				
Mean (SD)	46.80 months (31.22)	51.13 months (37.50)	42.12 months (23.56)	
**Previous ocular procedures, no. (%)**				
None	15 (30.6)	9 (36.0)	6 (25.0)	*P* = 0.46
AMG	25 (51.0)	13 (52.0)	12 (50.0)	
LKP/PKP	7 (14.3)	4 (16.0)	3 (12.5)	
SR+CFC	13 (26.5)	4 (16.0)	9 (37.5)	
Entropion correction	1 (2.0)	1 (4.0)	0 (0)	
**Cause of limbal stem cell deficiency, no. (%)**				
Alkaline burn	26 (53.1)	17 (70.8)	9 (36.0)	*P* = 0.06
Acid burn	9 (18.4)	4 (16.7)	5 (20.0)	
Thermal burn	8 (16.3)	1 (4.2)	7 (28.0)	
Rheumatism	4 (8.1)	2 (8.3)	2 (8.0)	
Explosive injury	2 (4.1)	0 (0.0)	2 (8.0)	
**Duration (mos) between injury and keratoplasty, no. (%)**				
Mean (SD)	44.2 ± 61.7	31.8 ± 39.6	57.3 ± 76.2	*P* = 0.05
≤6	10 (20.4)	0	4 (16.7)	
>6	39 (79.6)	25 (100.0)	20 (83.3)	
**Survival rate of grafts in different postoperative periods**				
1 year (%, sem)	79.59, 5.76	79.17%, 8.30	80%, 8.00	*P* = 0.52
3 years (%, sem)	70.52%, 6.66	64.86%, 14.17	75.79%, 8.61	
**LSCD Grade, no. (%)**				
Severe	7 (14.3)	3 (12.5)	4 (16)	*P* = 0.73
Total	42 (85.7)	21 (87.5)	21 (84)	

AMG, amniotic membrane transplantation; LK, lamellar keratoplasty; PKP, penetrating keratoplasty; SR, symblepharon release; CFC, conjunctiva flap covering.

^a^The Pearson chi-square (asymptotic 2-sided significance) was used for the gender, number of patients (eyes), indications for surgery, preoperatively, history of ocular surgery, duration time, survival rate, and LSCD grade.

### Surgical decision tree

The choice of surgical procedure for all patients was mainly based on slit lamp, confocal microscopy and anterior segment OCT results, including evaluation of corneal scars and corneal stroma. If complete corneal conjunctivalization prevents preoperative assessment of the corneal condition, a spare donor cornea should be prepared before surgery. The subconjunctival fibrovascular tissue was separated, exposing the cornea during the operation, to determine whether to add DALK surgery.

### Surgical technique

**FIGURE 1:**
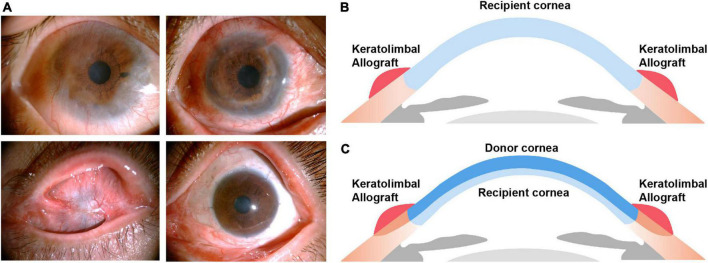
**(A)** Postoperative outcomes of KLAL transplantation in patients with severe bilateral limbal stem cell deficiency caused by chemical burns. Clinical photographs of the right eye of a 57-year-old male before KLAL-only transplantation (a), 12 months after KLAL-only transplantation (b) and the left eye of a 40-year-old female before KLAL transplantation combined with DALK (KLAL-DALK) (c), 12 months after KLAL-DALK (d). **(B,C)** The positioning of the graft in relation to angle structures. Keratolimbal allograft transplantation **(B)**. Keratolimbal allograft transplantation combined with deep anterior lamellar keratoplasty **(C)**. KLAL, keratolimbal allograft; DALK, deep anterior lamellar keratoplasty.

Two surgeons (Liqiang Wang and Yifei Huang) performed all surgeries at one center (Ophthalmology Department, PLA General Hospital), 40 under general anesthesia and 9 under local anesthesia. Details of the surgical technique for KLAL have previously been described (11, 21). Briefly, corneal clarity and corneal scars were assessed before or during surgery. If the cornea was clear, only KLAL transplantation was performed (KLAL-only) (Figure 1). A 360° limbal peritomy was performed, and symblepharon was addressed by excision and conjunctival recession at the limbus. A small conjunctival incision was made 2 mm above the limbus of the cornea to separate the subconjunctival fibrovascular tissue from the conjunctival epithelium. The superficial fibrovascular tissue over the corneal surface was removed using a combination of blunt and sharp dissection to create a smooth surface. The central cornea of two corneoscleral rims (stored at 4°C in Optisol for less than 5 days) was excised with a 7.5-mm or 8-mm trephine, and the remaining corneoscleral rim was stripped of excess scleral tissue 2–3 mm from the peripheral limbus. The anterior corneal edge overlying the recipient limbus was fixed with 10–0 nylon sutures. The graft conjunctiva and the recipient conjunctiva were sutured and fixed on the shallow sclera 4 mm from the peripheral limbus with 8–0 absorbable sutures. If the stroma was thinning or corneal scars were deep, DALK combined with KLAL transplantation (KLAL-DALK) was required (Figure 1) (22). Corneal trephination was done using a 7.5–8 mm trephine (Katena, Denville, NJ, USA) to three-fourths of the corneal depth, though the actual resulting trephination depth could be estimated to a certain degree only. Then lamellar dissection was initiated. In manual lamellar dissection, crescent blades and blunt dissection are usually used for lamellar dissection. Fresh KLAL donor tissue was prepared from cornea rims fixed to an artificial anterior chamber (Katena Products, Denville, NJ, USA), which allowed the processing of thin, ring-shaped donor grafts. The donor button was prepared with a diameter 0.5 mm larger than the bed. Then a lamellar dissection knife was used to dissect a thin, 360° KLAL donor (22). The Descemet membrane with or without 5–10% posterior stroma was then removed from the donor corneal button to match the host margin thickness. This was then placed on the bed and sutured using 10–0 nylon interrupted sutures. Damage to the microenvironment or the physical defense barrier of the ocular surface was significantly associated with failure. Meticulous ocular surface reconstruction was performed in patients with symblepharon and conjunctival sac narrowing.

### Postoperative management

Patients were given 5 mg dexamethasone intravenously for the first 24 h and cefuroxime for the first 3–5 days. The patients received prednisolone acetate tablets 40 mg daily for 1 week, which was gradually tapered; tobramycin and dexamethasone eye ointment (tobramycin 0.3%, dexamethasone 0.1%) 4 times daily for 1 week, which was replaced with 1% prednisolone acetate eye ointment 4 times daily for 2 weeks, which was gradually tapered; 0.5% levofloxacin eye ointment 4 times daily for 1 month; and 0.1% tacrolimus (FK506 eye drops) 4 times daily for long-term. Patients were asked to come to on-site follow-ups every 3–6 months.

### Statistical analysis methods

Statistical analysis was performed using GraphPad Prism 7 (GraphPad Software Inc., La Jolla, California, USA) and IBM SPSS Statistics 26.0 (International Business Machines Corporation, Armonk, New York, USA). The survival probability of KLALs was computed by Kaplan–Meier survival analysis. For comparisons of Kaplan–Meier curves, the log-rank (Mantel–Cox) test and the Gehan–Breslow–Wilcoxon test were used. Postoperative BCVA values were compared with the preoperative values by the Wilcoxon matched-pairs signed rank test. The weighted chi-square test was used to compare the success rates and the postoperative complication rates of the two procedures. Depending on the type of data and sample size, Pearson’s chi-square test or continuity correction was performed. For these comparisons, the significance level was set to 0.05.

## Results

The clinical details of all the patients are compiled in Table 1. There were a variety of LSCD etiologies. Alkali burns were the most common cause (26 eyes, 53.0%), followed by acid burns (9 eyes, 18.4%) and thermal burns (8 eyes, 16.3%) (Table 1).

### Success rate

**FIGURE 2:**
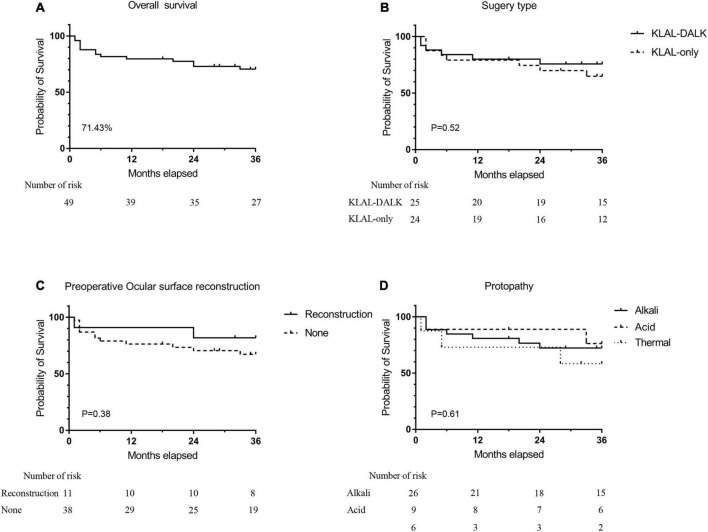
Kaplan–Meier survival analysis of graft survival with a stable corneal surface after KLAL transplantation. Survival analysis of grafts for all treated eyes (*n* = 14). Three years after surgery, 71.43% of eyes had a stable and improved corneal surface **(A)**. Grafts in eyes that underwent KLAL transplantation only (*n* = 24) had a similar survival curve as grafts in eyes that underwent KLAL transplantation with DALK (*n* = 25). Three years after surgery, the corneal surface was stable in 66.7% of eyes in the KLAL-only group and 76% of eyes in the KLAL-DALK group **(B)**. Survival analysis of grafts on the basis of preoperative etiology **(C)**, whether ocular surface reconstruction **(D)** was performed before KLAL transplantation, which showed no significant difference (log-rank test, *P* > 0.05). KLAL, keratolimbal allograft; DALK, deep anterior lamellar keratoplasty; LSCD, limbal stem cell deficiency.

At the final follow-up, 35 (71.43%) of the 49 eyes showed improved postoperative clinical outcomes (success or partial success). Sixteen (66.67%) eyes improved postoperatively in the KLAL-only group and 19 (76%) in the KLAL-DALK group. The improvement rate was not significantly different between the two groups (*P* = 0.47). Improvement at the 1-year follow-up was found in 39 (79.59%) of the 49 total eyes, 19 (79.17%) eyes in the KLAL-only group, and 20 (80%) in the KLAL-DALK group (Figure 2 and Supplementary material 1). Kaplan–Meier survival analysis showed that the 3-year survival probability of grafts among all eyes was 70.53 ± 10.89%, among eyes of the KLAL-only group was 64.86 ± 10.11%, and among eyes of the KLAL-DALK group was 75.79 ± 8.62% (*P* = 0.52) (Figure 2).

Kaplan–Meier survival analysis of grafts based on preoperative LSCD degree, etiology, time from injury to surgery, and whether PKP/LKP, ocular surface reconstruction, or amniotic membrane/oral mucosa transplantation was performed before KLAL revealed no significant risk factors associated with treatment failure during the available follow-up period (log-rank test, *P* > 0.05) (Figure 2 and Supplementary material 2).

### Vision

Given that LSCD patients’ visual acuity is mostly “finger counting,” “hand movement,” “light perception,” etc., it was hardly possible to perform a valid statistical analysis of their visual acuity (23, 24). Therefore, we adopted a method that could convert finger counting, hand motion, and light perception into the overall logarithm of the minimum angle of resolution visual acuity (LogMAR VA), which was proposed by Schulze-Bonse and Lange (“Counting Fingers” = 2, “Hand Motion” = 2.903, “Light Perception” = 3.204, “No Light Perception” = 3.505) (23, 24).

**FIGURE 3:**
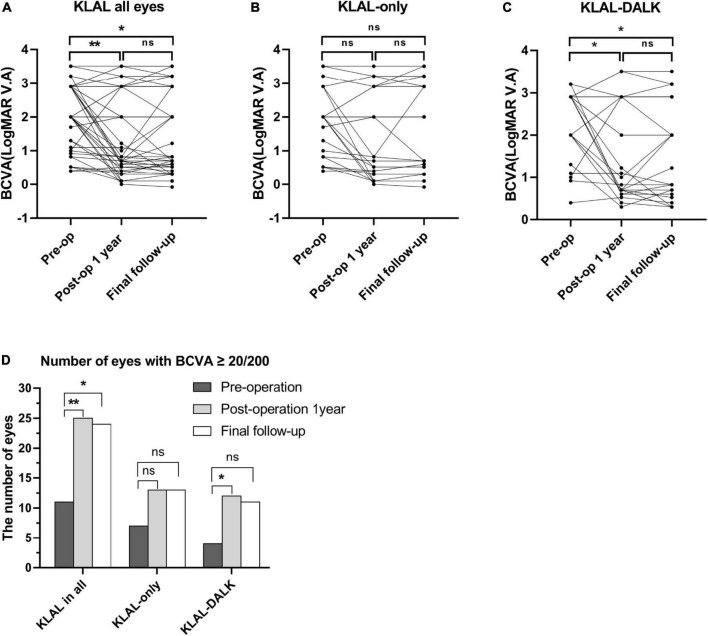
BCVA preoperatively and at the 1-year and final follow-up after KLAL transplantation in all eyes. In the whole patient cohort (*n* = 49), the BCVA at 1 year postoperation (*P* = 0.002) and at the final follow-up (*P* = 0.01) was significantly improved compared with that preoperation **(A)**. For the KLAL-only group (*n* = 24), the BCVA improved postoperatively but was not significantly different from that preoperatively (*P* = 0.06, 0.17) **(B)**. For the KLAL-DALK group (*n* = 25), the BCVA at 1 year postoperation (*P* = 0.02) and at the final follow-up (*P* = 0.03) was significantly improved compared with that preoperation **(C)**. The number of eyes with a BCVA ≥ 20/200 preoperatively, postoperatively, 1 year postoperatively and at the final follow-up **(D)**. Data are presented in dot plots including all individual points (*p < 0.05, **p < 0.01). A Wilcoxon matched-pairs signed rank test was used to compare the postoperative and preoperative BCVA values. BCVA, best corrected visual acuity; KLAL, keratolimbal allograft; DALK, deep anterior lamellar keratoplasty.

Visual acuity improvement was defined as a BCVA gain ≥ 2 lines. Among the 49 eyes, compared to preoperation, the number of eyes with improved overall visual acuity at 1 year postoperation and at the last follow-up was 33 (67.35%) and 34 (69.39%), respectively. In the KLAL-only group, 16 (66.67%) of the 24 eyes had improved visual acuity both at the 1-year postoperative period and at the final follow-up. In the KLAL-DALK group, 17 (68%) and 18 (72%) of 25 eyes had visual acuity improvement at 1 year postoperation and at the final follow-up, respectively (Figure 3).

The preoperative baseline visual acuity in all eyes in the KLAL group was 2.00 (2.90∼1.20) (median with interquartile range), and that in the KLAL-only group and KLAL-DALK group was 2.00 (2.67∼0.87) and 2.90 (2.90∼1.65), respectively. For the KLAL-only group, there was no statistically significant improvement in visual acuity at 1 year postoperation [0.82 (2.67∼0.33), *P* = 0.06] or at the last follow-up [1.22 (2.90∼0.52), *P* = 0.17]. However, the results in the KLAL-DALK group suggested that visual acuity at 1 year postoperation [1.10 (2.90∼0.70), *P* = 0.02] and at the last follow-up [2.0 (2.90∼0.65), *P* = 0.03] showed a statistically significant improvement compared with preoperation.

The analysis of the overall change in vision with time showed a progressive increase in the cumulative proportion of eyes with a BCVA of 20/200 (logMAR BCVA = 1) or better (Figure 3). The proportion of eyes with a BCVA ≥ 20/200 among all eyes that underwent KLAL transplantation increased from 11 eyes (22.45%) preoperatively to 25 eyes (51.02%) (*P* < 0.01, pre- vs. 1 year) at the 1-year follow-up and to 24 eyes (48.98%) (*P* = 0.01, pre- vs. last) at the last follow-up. The proportion of eyes with a BCVA ≥ 20/200 in the KLAL-only group increased from 7 eyes (28.0%) preoperatively to 13 eyes (54.17%) (*P* = 0.14, pre- vs. 1 year/last) at both the 1-year follow-up and the last follow-up, but this increase was not statistically significant (*P* = 0.19, pre- vs. 1 year vs. last). The proportion of eyes with a BCVA ≥ 20/200 in the KLAL-DALK group increased significantly, from 4 eyes (16.0%) preoperatively to 12 eyes (48.0%) (*P* = 0.03, pre- vs. 1 year) after 1 year of follow-up and to 11 eyes (44.0%) (*P* = 0.06, pre- vs. last) at the last follow-up.

### Complications

A total of 49 eyes were enrolled in this study, and 17 eyes (34.69%) had postoperative complications (4 eyes had 2 complications). The incidence of corneal ulcers was 18.37% (9 eyes), followed by glaucoma at 8.16% (4 eyes) and corneal perforation at 6.12% (3 eyes). Among the 24 eyes in the KLAL-only group, 8 eyes (33.33%, 4 eyes had 2 complications) had complications. The incidence of corneal ulcers was 29.17% (7 eyes), the incidence of glaucoma was 8.33% (2 eyes), and the incidence of limbal implant cysts and leukoplakia was 4.17% (1 eye). In the KLAL-DALK group, 9 of the 25 eyes (36%) had complications. The incidence of corneal ulcers, glaucoma, and corneal perforation was each 8.0% (2 eyes), and the incidence of keratitis, cataract, and corneal pannus (Stromal rejection) was each 4.0% (1 eye) (Table 2).

**TABLE 2:** The incidence of complications in the KLAL-only group and KLAL-DALK group.

Postoperative complication	Total (%)	KLAL-only (%)	KLAL-DALK (%)	Subgroup
				*P*-value^a^
Corneal ulcer	9 (18.37)	7 (29.2)	2 (8.0)	0.06
Glaucoma^b^	4 (8.2)	2 (8.3)	2 (8.0)	0.97
Corneal perforation	3 (6.1)	1 (4.2)	2 (8.0)	0.58
Cataract	1 (2.0)	0 (0.0)	1 (4.0)	0.32
Corneal fibrovascular pannus	1 (2.0)	0 (0.0)	1 (4.0)	0.32
Keratitis	1 (2.0)	0 (0.0)	1 (4.0)	0.32
Limbal implantation cyst	1 (2.0)	1 (4.2)	0 (0.0)	0.30
Corneal opacity	1 (2.0)	1 (4.2)	0 (0.0)	0.30
Total	17*	8*	9	0.8446

*In KLAL-only group, 4 eyes had 2 complications. A total of 49 eyes were included: 24 eyes in the KLAL-only group and 25 eyes in the KLAL-DALK group.

^a^The Pearson chi-square (asymptotic 2-sided significance) was used for the postoperative complication.

^b^Glaucoma included 2 eyes of progressing glaucoma (both in KLAL-only group) and 2 eyes of postoperative new-onset developed glaucoma (both KLAL-DALK group).

Corneal ulcers occurred in 7 eyes in the KLAL-only group and 2 eyes in the KLAL-DALK group, accounting for 29.17% (7/24 eyes in the KLAL-only group) and 8% (2/25 eyes in the KLAL-DALK group) of eyes with postoperative complications, respectively. The incidence of corneal ulcers in the KLAL-DALK group was less than that in the KLAL-only group (*P* = 0.06).

There was no difference in the allograft rejection rate between KLAL and KLAL-DALK (*P* = 0.76). In the KLAL-only group, 4 eyes experienced KLAL rejection (16.67%), 3 eyes had corneal ulcers, and 1 eye had corneal perforation. In the KLAL-DALK group, 5 eyes experienced rejection after transplantation (20%, including 1 corneal stromal rejection), 2 eyes had corneal ulcers, and 2 eyes had corneal perforation.

## Discussion

The reconstruction of the ocular surface in eyes with severe bilateral LSCD remains one of the most challenging problems in ophthalmology. Bilateral LSCD presents an entirely different set of challenges than unilateral LSCD with regard to visual rehabilitation. In addition to LSCD, there is damage to the adnexa, including the eyelids, cilia, lacrimal and meibomian glands, and conjunctiva. Appropriate management of adnexal pathology prior to ocular surface reconstruction is therefore imperative (25, 26). Recent studies have suggested that adnexal problems, such as eyelid abnormalities, keratinization and symblepharon, are important prognostic factors adversely affecting the outcome of surface reconstruction (1, 27). In this study, we preserved as much conjunctival tissue as possible during surgery, and the structure and function of the forniceal conjunctiva and conjunctival sac were restored after surgery in all eyes.

In the presence of significant corneal stromal scarring, transplantation of KLAL alone will not result in adequate visual rehabilitation. In such cases, KLAL transplantation can also be combined with PKP or DALK either simultaneously or sequentially, resulting in remodeling of the corneal scars. Previous studies with short-term follow-up echoed the findings of rapid surface reconstruction and visual improvement (13, 28). However, when PKP is performed, even with surface stabilization, because of the prior history of inflammation and a vascularized host bed, the graft remains high-risk and can be rejected. Later studies with longer follow-up, however, revealed a significant decline in allograft survival and visual acuity, especially in KLAL transplantation combined with PKP (15, 16, 18, 27). The success rate of KLAL transplantation declines from 75 to 80% (13, 28, 29) after 1 year to 50% after 3 years of follow-up (30). Eyes undergoing allograft transplantations performed simultaneously with PKP fared worse, and the survival of grafts was low (15, 16). Solomon et al. reported a large series of KLAL transplantations in 39 eyes, 23 of which underwent simultaneous PKP, with a mean follow-up period of almost 3 years (16). Survival analysis showed a progressive decline in KLAL survival (a 77% survival rate at 1 year and only 24% at 5 years) and ambulatory vision (77% at 1 year and 45% at 5 years) with time. In addition, in eccentric PKP with a section of limbus, 18 (72%) of 25 grafts failed. Performing PKP concomitantly with KLAL transplantation was found to decrease the visual outcome compared with performing KLAL transplantation alone. Other long-term studies showed similar findings of partial and limited success of allograft transplantation (16, 27). Particularly in eyes with aniridia and Stevens-Johnson syndrome, allografts performed poorly, despite maximal medical and immunosuppressive treatments and were associated with significant complications (16, 19, 27). Some studies reported that two-stage surgery had good outcomes (31, 32), but others considered successful KLAL to carry a high risk of subsequent PKP failure (33). These studies cannot be directly compared because of insufficient information, but one thing is sure: there is a delay of 6–12 months before the second surgery can be performed, which in turn postpones the visual rehabilitation. A second surgical procedure can also result in a loss of a substantial number of stem cells, leading to increased stress on the transplanted stem cell population. DALK is equivalent to PKP in the outcome measure of BCVA, while DALK is superior for the preservation of endothelial cell density (34).

The major advantage of DALK is that the host corneal endothelium is kept in place, and thus endothelial immune graft rejection cannot occur after DALK, which may simplify the long-term management of DALK eyes compared with PKP eyes (11, 21, 22). Thus, only epithelial, subepithelial, or stromal immune reactions can occur. In our current study, only one patient (No. 3 eye) occurred stromal rejection 6 months after surgery. The patients with stromal rejection were treated with dexamethasone 0.1% eye drops 6 times a day for 14 days and then all episodes resolved within 6 months after the onset. Although the host epithelial cells and keratocytes repopulate the donor tissue, as late rejections after 3–4 years have been reported (35). It seems that with time the risk of epithelial, subepithelial, and stromal rejection episodes might decrease. However, up to now, no stromal rejection has occurred over 1 year in our study. We will pay close attention to it in the future follow-up. As an extraocular procedure, DALK has important theoretical safety advantages, and it is a good option for visual rehabilitation of corneal disease in patients whose endothelium is not compromised (36). In patients with unilateral, late-stage, severe LSCD, simultaneous or staged DALK combined with autologous limbal transplantation can restore a stable ocular surface (22, 37). However, the long-term prognosis of allograft KLAL transplantation simultaneously combined with DALK for bilateral severe LSCD has not been reported. The findings of this study suggest that KLAL transplantation combined with DALK is effective in ocular surface and corneal restoration and visual improvement in eyes with bilateral LSCD in the presence of corneal leukoma and normal endothelial function.

The findings from a recent meta-analysis showed that an improvement in the ocular surface was achieved in 57.8% of eyes (95% CI, 49.0–66.1%) after KLAL transplantation (38), which was in agreement with the findings of this study, which showed that the 3-year survival probability of grafts for all eyes was 70.53 ± 10.89%, for KLAL-only group eyes was 64.86 ± 10.11%, and for KLAL-DALK group eyes was75.79 ± 8.62% (*P* = 0.52) (Figure 2).

KLAL transplantation simultaneously with DALK (KLAL-DALK) not only addresses the fact that KLAL-only cannot treat corneal stromal scars and stromal thinning but also avoids the risk of endothelial graft rejection after KLAL transplantation simultaneously with PKP (KLAL-PKP). On the one hand, KLAL-DALK (proportion of BCVA ≥ 20/200; *P* = 0.14, pre- vs. 1 year) rapidly reconstructed visual acuity in patients who presented with corneal opacity in the short term compared with KLAL-only (proportion of eyes with BCVA ≥ 20/200; *P* = 0.19) and reduced the incidence of corneal ulcers (KLAL-only 29.17%, KLAL-DALK 8.00%; *P* = 0.07). Perhaps the fact that extra healthy DALK stroma tissue is used in the combined KLAL-DALK procedure gives the diseased, thinned cornea more bulk so that it is tectonically stronger and can withstand infection or inflammation better after operation. On the other hand, KLAL-DALK had similar short-term visual outcomes as KLAL-PKP, which were a high rate of failure and visual loss during long-term follow-up. Fortunately, the rate of surgical failure was not higher than that of the KLAL-only group under the same postoperative medications. Postoperative glaucoma is a serious complication after corneal surgery. The incidence of glaucoma after KLAL-DALK was 8.0%, similar to the 8.3% in the KLAL-only group and lower than the 43% reported in studies by Solomon et al. (16). Here’s the thing to notice, there is a lack of effective non-invasive methods to evaluate the condition of endothelial layer in patients with corneal opacity at present. Especially that assessment of the health of the underlying endothelium in the presence of significant corneal opacity is not possible clinically. Confocal microscopy, if available, can be attempted to evaluate the endothelium (39, 40). Additionally, anterior segment OCT is another option which allows for the imaging of all corneal layers and structures in great detail (41, 42). If the endothelium layer cannot be evaluated preoperatively, a clear residual bed cannot be obtained during operation, the lamellar graft continues to remain edematous following surgery, one should be prepared to convert the procedure to a full-thickness penetrating keratoplasty (22).

There are some limitations to this study, which was a retrospective, non-randomized controlled study. Eyes with worse central scarring and corneal thinning needing DALK are eyes with more severe disease at baseline, so there may have been a disease-related bias, and patients in the KLAL-DALK group may have had more severe preoperative corneal damage and corneal scarring than those in the KLAL-only group. Since there were diverse etiologies and disease baselines in both groups, it was difficult to directly compare them. Even so, the final outcome of KLAL-DALK with more severe disease before surgery reflected the good potential of this simultaneous combined surgery.

Due to the allogeneic nature of limbal allografts, rejection is the main risk for failure, and these patients require not only local but also systemic, and often long-term, immunosuppression, which can lead to frequent and sometimes serious side effects such as anemia, renal and liver function impairment, and hyperglycemia (1, 25, 43, 44). Instead of using systemic immunosuppressants, we chose to use short-term courses of high-dose steroids and tacrolimus (FK506 eye drops) locally in this study. On the one hand, this protocol can avoid a number of systemic side effects, including nephrotoxicity, hypertension hyperlipidemia (45, 46), infections, leucopaenia, anemia, and gastrointestinal disturbances (47). On the other hand, FK506 eye drops are as effective as Mycophenolate mofetil (MMF) and the systematic using CSA. Meanwhile, the using FK506 were considered to be promising management to prevent rejection in high-risk penetrating keratoplasty in the present study (48). Furthermore, Yu et al. revealed that Topical tacrolimus (FK506) was more effective than topical cyclosporine A (CsA) at reducing the 1-year graft rejection rate (48, 49). In our current study, no patient experienced side effects related to FK506 eye drops. Therefore, we believe that using FK506 eye drops instead of systemic immunosuppression therapy is safe and effective. In addition, the patients received potassium (potassium chloride) to support the systemic use of hormones 3 times daily (0.5 g/time), calcium (calcium carbonate D3) 600 mg daily and a proton pump inhibitor (omeprazole) 20 mg/dose twice daily. There were no hormone-related complications in any patients.

In conclusion, we recommend the DALK procedure along with allogeneic KLAL transplantation for the management of patients with bilateral severe LSCD with corneal scars and compensable corneal endothelial function. This surgery, when performed in the late stage following ocular injury that is in a stable state, results in successful reconstruction of the ocular surface with good improvement in visual acuity. Meticulous restoration of the ocular surface defenses together with a combined immunosuppressive regimen is a prerequisite to improve the success rate and long-term visual outcome of all KLAL-related surgeries.

## Conclusion

In this study with a long follow-up duration, the success rate of were similar between the KLAL-only group and KLAL-DALK group. Compared with the KLAL-only group, the KLAL-DALK group was able to rapidly reconstruct vision in a short period, without an additional increase of allograft rejection and other complications incidence rate. Simultaneous DALK combined with KLAL transplantation is an effective option to restore a stable ocular surface and visual acuity rapidly in patients with bilateral, late-stage, severe LSCD.

## Data availability statement

The original contributions presented in the study are included in the article/Supplementary material, further inquiries can be directed to the corresponding author/s.

## Ethics statement

The studies involving human participants were reviewed and approved by the Chinese PLA General Hospital Ethical Board Committee. The patients/participants provided their written informed consent to participate in this study.

## Author contributions

LW and YH participated in the surgical procedure. ZL and KY participated in data collation, data arrangement, manuscript writing, and manuscript revision. YZ, TW, HZ, QY, and QW participated in data collation. All authors contributed to the article and approved the submitted version.
