# Supplementary material for: Outcome of keratolimbal allograft transplantation with deep anterior lamellar keratoplasty for bilateral limbal stem cell deficiency
Source: Front Med (Lausanne). 2022 Nov 15;9:986194. doi: 10.3389/fmed.2022.986194 (PMC9705574; doi:10.3389/fmed.2022.986194)
Supplement: Supplementary file 1 [file Data_Sheet_1.ZIP › Supplement1.docx]

| **Patient** | **Age**  **Range** | **Previous surgery** | **Pre V.A** | **LSCD Grade** | **Time from iujury（mon）** | **Sugery**  **Type** | **Adverse evets** | **V.A. 1year** | **Result**  **(1 year)** | **V.A. last follow-up** | **Result**  **last follow-up** | **Post-graft surgery** |
| --- | --- | --- | --- | --- | --- | --- | --- | --- | --- | --- | --- | --- |
| **1** | **34** | **AM** | **CF** | **total** | **24** | **KLAL** | **n** | **CF** | **Partially success** | **CF** | **Partially success** | **n** |
| **2** | **23** | **OSR** | **LP** | **total** | **24** | **KLAL** | **CFM; G** | **HM** | **partical success** | **NLP** | **Failure** | **OSR; LMR; ECP; TCP** |
| **3** | **23** | **OSR; AM; PK; LMR** | **HM** | **total** | **216** | **KLAL＋DALK** | **SR** | **HM** | **Partially success** | **HM** | **Partially success** | **OSR; LMR** |
| **4** | **12** | **LK** | **CF** | **total** | **120** | **KLAL** | **n** | **CF** | **partical success** | **0.2** | **Partially success** | **CE+IOL** |
| **5** | **26** | **OSR; AM; LMR** | **CF** | **total** | **24** | **KLAL** | **n** | **HM** | **Partially success** | **HM** | **Partially success** | **n** |
| **6** | **26** | **OSR; AM; LK** | **HM** | **total** | **24** | **KLAL＋DALK** | **n** | **0.06** | **Partially success** | **0.5** | **Partially success** | **n** |
| **7** | **48** | **n** | **CF** | **total** | **2** | **KLAL** | **n** | **1** | **Success** | **1.2** | **Success** | **n** |
| **8** | **43** | **OSR** | **0.12** | **severe** | **13** | **KLAL＋DALK** | *** (surgery)** | **0.15** | **Success** | **0.15** | **Success** | **CE+IOL** |
| **9** | **52** | **AM** | **CF** | **total** | **11** | **KLAL＋DALK** | **n** | **0.2** | **Success** | **0.5** | **Success** | **n** |
| **10** | **33** | **n** | **0.4** | **total** | **36** | **KLAL＋DALK** | **n** | **0.3** | **Success** | **0.4** | **Success** | **n** |
| **11** | **57** | **n** | **0.3** | **total** | **8** | **KLAL** | **n** | **0.5** | **Success** | **0.5** | **Success** | **n** |
| **12（B）s** | **53** | **AM** | **0.1** | **total** | **11** | **KLAL＋DALK** | **G (suegry)** | **HM** | **Partially success** | **NLP** | **Partially success** | **TCP** |
| **13 d** | **53** | **AM** | **HM** | **total** | **13** | **KLAL＋DALK** | **G (drug)** | **0.25** | **Partially success** | **0.15** | **Partially success** | **n** |
| **14** | **44** | **AM; PK** | **CF** | **total** | **13** | **KLAL＋DALK** | **CU (suegry)** | **HM** | **Failure** | **HM** | **Failure** | **OSR; LMR** |
| **15** | **27** | **OSR** | **CF** | **total** | **14** | **KLAL＋DALK** | **CP**  **(suegry)** | **0.1** | **Failure** | **CF** | **Failure** | **OSR; LMR** |
| **16** | **37** | **AM** | **0.08** | **total** | **5** | **KLAL＋DALK** | **CU (suegry)** | **0.08** | **Failure** | **0.15** | **Failure** | **CF** |
| **17** | **43** | **AM; LK** | **HM** | **total** | **84** | **KLAL** | **CU; CO** | **0.8** | **Failure** | **0.8** | **Failure** | **CF; LMR** |
| **18** | **48** | **AM** | **NLP** | **severe** | **2** | **KLAL** | **n** | **NLP** | **Partially success** | **NLP** | **Partially success** | **n** |
| **19** | **37** | **LK; AM; OSR; LMR** | **0.02** | **total** | **84** | **KLAL** | **n** | **CF** | **Partially success** | **CF** | **Partially success** | **n** |
| **20** | **31** | **n** | **0.15** | **total** | **60** | **KLAL** | **n** | **0.8** | **Success** | **0.5** | **Success** | **n** |
| **21** | **35** | **n** | **CF** | **total** | **4** | **KLAL** | **n** | **0.8** | **Success** | **0.8** | **Success** | **n** |
| **22** | **40** | **OSR; LMR** | **CF** | **total** | **14** | **KLAL＋DALK** | **n** | **0.4** | **Success** | **0.5** | **Success** | **n** |
| **23** | **50** | **AM** | **CF** | **total** | **12** | **KLAL** | **G (suegry)** | **HM** | **Failure** | **NLP** | **Failure** | **TCP** |
| **24** | **44** | **LK; AM; OSR; LMR** | **HM** | **total** | **96** | **KLAL＋DALK** | **n** | **CF** | **Success** | **CF** | **Success** | **n** |
| **25** | **52** | **n** | **LP** | **total** | **1** | **KLAL＋DALK** | **n** | **HM** | **Partially success** | **HM** | **Partially success** | **n** |
| **26** | **34** | **AM** | **HM** | **severe** | **7** | **KLAL** | **CU (suegry)** | **NLP** | **Failure** | **LP** | **Failure** | **LMR; AM** |
| **27** | **36** | **AM** | **0.3** | **total** | **6** | **KLAL** | **n** | **0.8** | **Success** | **0.8** | **Success** | **n** |
| **28** | **29** | **n** | **HM** | **total** | **144** | **KLAL＋DALK** | **n** | **0.2** | **Partially success** | **0.2** | **Partially success** | **n** |
| **29(B) d** | **49** | **AM** | **HM** | **total** | **2** | **KLAL＋DALK** | **n** | **TA** | **Partially success** | **LP** | **Partially success** | **n** |
| **30 s** | **49** | **AM; TR** | **0.3** | **severe** | **9** | **KLAL** | **CU; LIC** | **0.4** | **Partially success** | **0.25** | **Partially success** | ***** |
| **31** | **39** | **n** | **CF** | **severe** | **204** | **KLAL＋DALK** | **Keratitis (drug)** | **0.2** | **Failure** | **CF** | **Failure** | **n** |
| **32** | **62** | **n** | **0.1** | **total** | **9** | **KLAL** | **CU** | **0.15** | **Failure** | **HM** | **Failure** | **n** |
| **33** | **30** | **Cf** | **CF** | **total** | **15** | **KLAL＋DALK** | **n** | **HM** | **Partially success** | **HM** | **Partially success** | **n** |
| **34** | **60** | **n** | **0.4** | **severe** | **108** | **KLAL** | **n** | **0.4** | **Success** | **0.25** | **Success** | **n** |
| **35** | **28** | **n** | **CF** | **total** | **4** | **KLAL** | **CP**  **(suegry)** | **CF** | **Failure** | **CF** | **Failure** | **PK; LT; TR** |
| **36** | **56** | **AM; LT; TR** | **TA** | **total** | **12** | **KLAL** | **n** | **NLP** | **Partially success** | **HM** | **Failure** | **n** |
| **37** | **24** | **PK** | **CF** | **total** | **276** | **KLAL＋DALK** | **n** | **CF** | **Partially success** | **CF** | **Partially success** | **n** |
| **38** | **20** | **AM; ECP; CE; Cf** | **HM** | **total** | **10** | **KLAL＋DALK** | **n** | **0.2** | **Partially success** | **0.06** | **Partially success** | **n** |
| **39** | **64** | **n** | **HM** | **total** | **15** | **KLAL** | **n** | **0.15** | **Partially success** | **0.2** | **Partially success** | **n** |
| **40** | **34** | **OSR; AM; LMR** | **0.05** | **total** | **48** | **KLAL＋DALK** | **n** | **0.5** | **Partially success** | **0.2** | **Partially success** | **n** |
| **41** | **55** | **n** | **0.1** | **severe** | **72** | **KLAL＋DALK** | **CP (suegry)** | **HM** | **Partially success** | **CF** | **Failure** | **LT; rg** |
| **42** | **47** | **AM; Cf** | **LP** | **total** | **34** | **KLAL＋DALK** | **n** | **0.25** | **Failure** | **0.25** | **Failure** | **n** |
| **43** | **35** | **LK; AM; OSR; LMR** | **HM** | **severe** | **15** | **KLAL＋DALK** | **n** | **0.01** | **Partially success** | **0.01** | **Partially success** | **n** |
| **44** | **54** | **AM** | **0.02** | **severe** | **2** | **KLAL** | **GR** | **CF** | **Success** | **CF** | **Failure** | **LK** |
| **45** | **49** | **AM** | **0.05** | **severe** | **12** | **KLAL** | **n** | **0.3** | **Success** | **0.3** | **Success** | **n** |
| **46** | **34** | **EC** | **CF** | **severe** | **8** | **KLAL** | **n** | **0.3** | **Success** | **0.3** | **Success** | **n** |
| **47** | **35** | **ACP** | **HM** | **total** | **0.5** | **KLAL＋DALK** | **n** | **TA** | **Partially success** | **TA** | **Partially success** | **n** |
| **48** | **16** | **n** | **0.01** | **total** | **108** | **KLAL＋DALK** | **n** | **0.2** | **Partially success** | **0.3** | **Partially success** | **n** |
| **49** | **18** | **n** | **0.15** | **total** | **132** | **KLAL** | **n** | **0.2** | **Success** | **0.2** | **Success** | **n** |

**Partial LSCD** is defined as incomplete conjunctivalization of the corneal surface and the presence of residual limbal and consequent corneal epithelial cells.

**Total LSCD** is defined as conjunctivalization of the entire corneal surface due to a complete loss of corneal epithelial stem/progenitor cells.

**ACP** anterior chamber penetration, **AM** amniotic membrane graft, **(B)** bilateral injury, **CE** cataract extraction, **Cf** conjunctival flap, **CF** counting fingers, **CFM** corneal flap melting, **CO** corneal opacity, **CP** corneal perforation, **CU** corneal ulcer, **DALK** deep anterior lamellar keratoplasty, **EC** eyelash coagulation, **ECP** endoscopic cyclophotocoagulation, **ED** epithelial defect, **ER** eyelid reconstruction, **G** glaucoma, **HM** hand movement, **IOL** i[ntraocular](https://cn.bing.com/dict/search?q=intraocular&FORM=BDVSP6&cc=cn) [lens](https://cn.bing.com/dict/search?q=lens&FORM=BDVSP6&cc=cn), **KLAL** keratolimbal allograft, **LIC** limbal implantation cyst, **LK** lamellar keratoplasty, **LMR** labial mucosa transplantation, **LP** light perception, **LT** limbal transplantation, **n** none, **NLP** no light perception, **OSR** ocular surface reconstruction, **PK** penetrating keratoplasty, **rg** re-grafting, **SC** strabismus correction, **SR** stromal rejection, **TA** Tarsorrhaphy, **TCP** Transscleral cyclophotocoagulation, **TR** tarsorrhaphy, **VA** best corrected visual acuity,  ***** amblyopia, cataract, corneal graft failure or other ocular problems not related to the ocular surface
