# Supplementary material for: Outcome of keratolimbal allograft transplantation with deep anterior lamellar keratoplasty for bilateral limbal stem cell deficiency
Source: Front Med (Lausanne). 2022 Nov 15;9:986194. doi: 10.3389/fmed.2022.986194 (PMC9705574; doi:10.3389/fmed.2022.986194)
Supplement: Supplementary file 1 [file Data_Sheet_1.ZIP › Supplement2.docx]

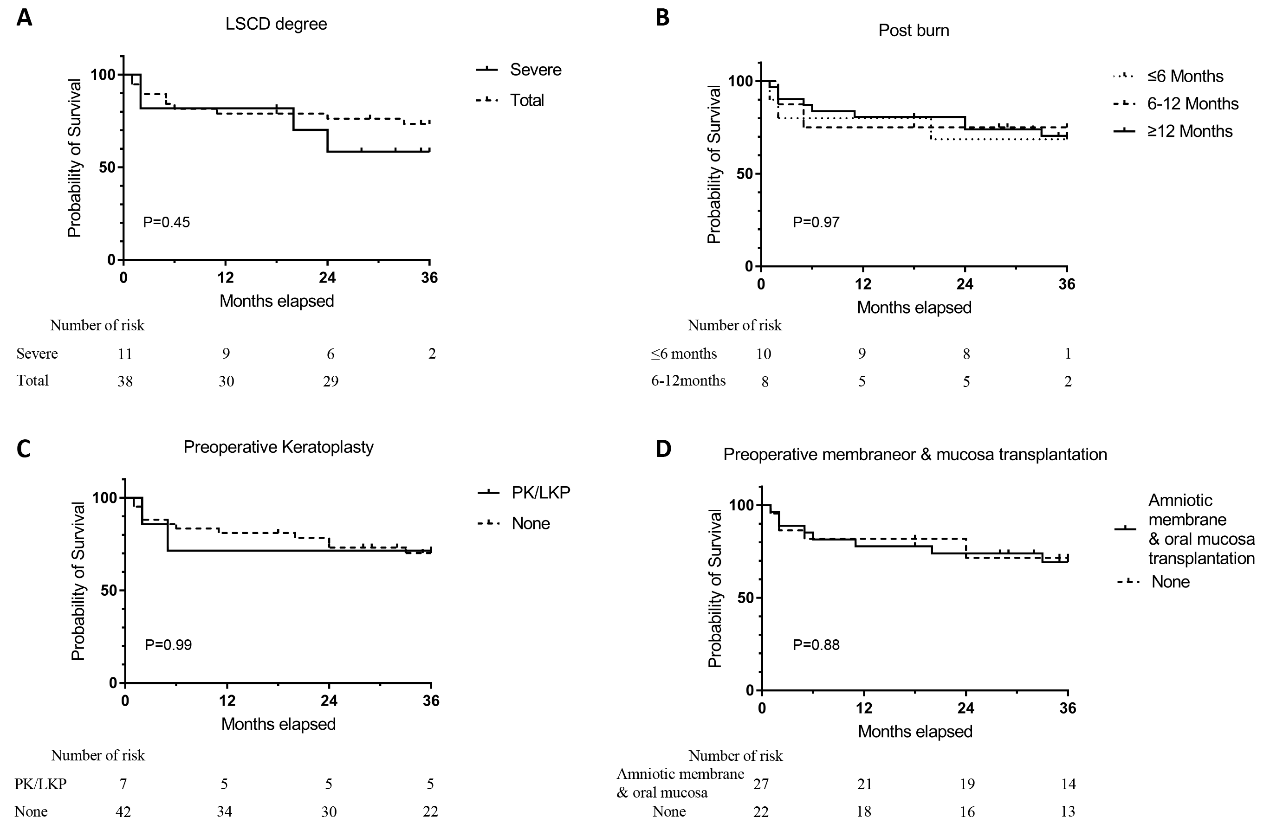
 Supplement 2. Survival analysis of grafts on the basis of preoperative LSCD degree (A), time from injury to surgery (B) and whether PKP/LKP (C), or amniotic membrane/oral mucosa transplantation(D) was performed before KLAL transplantation, which showed no significant difference (log-rank test, P>0.05). KLAL, keratolimbal allograft; DALK, deep anterior lamellar keratoplasty; LSCD, limbal stem cell deficiency.
