# Supplementary figures and images for: Outcome of keratolimbal allograft transplantation with deep anterior lamellar keratoplasty for bilateral limbal stem cell deficiency
Source: Front Med (Lausanne). 2022 Nov 15;9:986194. doi: 10.3389/fmed.2022.986194 (PMC9705574; doi:10.3389/fmed.2022.986194)

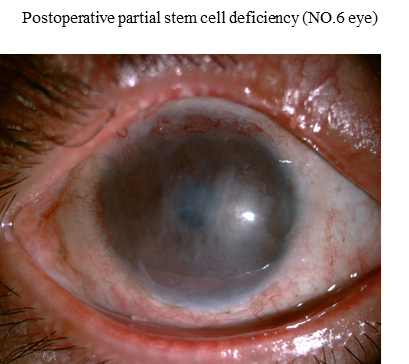

Supplement: Supplementary file 1 [file Data_Sheet_1.ZIP › Supplement3.docx]
